# A phase 1 study evaluating the pharmacokinetics, safety and tolerability of repeat dosing with a human IL-13 antibody (CAT-354) in subjects with asthma

**DOI:** 10.1186/1471-2466-10-3

**Published:** 2010-01-08

**Authors:** Dave Singh, Binita Kane, Nestor A Molfino, Raffaella Faggioni, Lorin Roskos, Ashley Woodcock

**Affiliations:** 1 Medicines Evaluation Unit, University Hospital of South Manchester Foundation Trust, University of Manchester, Manchester, UK; 2 Respiratory and Inflammation, MedImmune LLC, Gaithersburg, Maryland, USA

## Abstract

**Background:**

IL-13 has been implicated in the development of airway inflammation and hyperresponsiveness. This study investigated the multiple-dose pharmacokinetics and safety profile of human anti-IL-13 antibody (CAT-354) in adults with asthma.

**Methods:**

This was a multiple-dose, randomised, double-blind, placebo-controlled phase 1 study in asthmatics (forced expiratory volume in 1 second [FEV_1_] ≥ 80% predicted). Subjects were randomised to receive three intravenous infusions of CAT-354 (1 mg/kg, 5 mg/kg or 10 mg/kg) or placebo at 28-day intervals. Blood samples were taken for pharmacokinetic measurements. Safety was assessed by adverse events, vital signs, ECGs, laboratory and pulmonary function parameters.

**Results:**

Twenty-three subjects (aged 21-60 years, FEV_1 _88-95% predicted) received ≥ 1 dose of study medication. The half-life of CAT-354 was 12-17 days and was dose-independent. The maximum serum concentration and area under the curve were dose-dependent. Clearance (2.2-2.6 mL/day/kg) and volume of distribution (44-57 mL/kg) were both low and dose-independent. The observed maximum serum concentration after each dose increased slightly from dose 1 through dose 3 at all dose levels, consistent with an accumulation ratio of 1.4 to 1.7 for area under the curve. Most adverse events were deemed mild to moderate and unrelated to study medication. One SAE was reported and deemed unrelated to study drug. There were no effects of clinical concern for vital signs, ECG, laboratory or pulmonary parameters.

**Conclusions:**

CAT-354 exhibited linear pharmacokinetics and an acceptable safety profile. These findings suggest that at the doses tested, CAT-354 can be safely administered in multiple doses to patients with asthma.

**Trial registration:**

NCT00974675.

## Background

Asthma is characterised by variable airflow obstruction and airway hyperresponsiveness (AHR) in association with airway inflammation [1]. Inhaled corticosteroids (ICS) are currently the first-line anti-inflammatory treatment for persistent asthma [1]. However, many asthma patients remain symptomatic despite ICS therapy [2,3]. Alternative anti-inflammatory therapies are needed in asthma.

T helper-2 (TH-2) lymphocytes release cytokines, including IL-4, IL-5 and IL-13, that have a range of actions, including eosinophil activation and immunoglobulin secretion from B cells. Clinical studies have shown that asthma is associated with TH-2 inflammation [4-6]. Targeting the cytokines involved in TH-2 inflammation may therefore be an effective therapeutic strategy.

IL-13 levels are increased in the airways of patients with asthma [7,8]. Of particular importance is the finding that IL-13 positive cells are present within the airway smooth muscle and expressed predominantly by mast cells, suggesting that IL-13 plays a pivotal role in mast cell-airway smooth muscle interactions [9]. The genes encoding for IL-13 and IL-4 are both located on the cytokine cluster on chromosome 5q31. These TH-2 cytokines share some structural similarities, and both exert their actions through the IL-4Rα/IL-13Rα1 receptor complex; therefore, these cytokines have overlapping functions. IL-4 also exerts independent effects through the IL-4Rα/γ receptor. However, animal models suggest a dominant role for IL-13 in the pathophysiology of allergic inflammation, as IL-13 causes AHR, eosinophilic inflammation and mucus hypersecretion [10-14]. Antagonising the function of IL-13 in asthma may be a therapeutically effective strategy.

CAT-354 is a high affinity, human monoclonal IgG4 antibody that specifically binds to and neutralises IL-13. This study aimed to assess the pharmacokinetics, tolerability and safety of repeated doses of CAT-354 in subjects with mild to moderate asthma.

## Methods

### Subject eligibility

This study was conducted at two UK sites: the Medicines Evaluation Unit and the Chiltern clinical research unit. Ethics approval was obtained at both sites and the study was conducted in accordance with ICH Good Clinical Practice guidelines and in compliance with the 2000 Declaration of Helsinki. All subjects provided written informed consent prior to the performance of any study-specific procedures.

Subjects aged 18 to 60 years with a physician diagnosis of asthma were eligible to participate in this study. Female subjects were either postmenopausal (no menstrual period for a minimum of 1 year) or surgically sterilised. Subjects had to have a forced expiratory volume in 1 second (FEV_1_) of ≥ 80% of predicted normal and be well controlled on ICS and short-acting β_2_-agonists (SABA) only with no change in the dose of ICS for 3 months prior to the study. Subjects were also required to not have smoked in the previous year and have a smoking history of ≤ 10 pack years.

Exclusion criteria were an asthma exacerbation requiring hospitalisation within 3 years of the study, a history of any active disease other than eczema, seasonal allergy which was expected to start before the last dose of study material, poorly controlled asthma defined as SABA > 6 times/day on any one day or > 3 times/day on six or more days within the 2 weeks prior to the study, previous treatment with any other asthma medications within 6 months of the study, treatment for atopic symptoms except eczema within the previous 4 weeks, any acute illness in the prior 2 weeks, a lower respiratory tract infection within 4 weeks, previous treatment with a monoclonal antibody or related protein and participation in another study within 3 months (or 5 half-lives of the investigational product). Participants had to have a medical history negative for alcohol or substance abuse and no clinically significant ECG or clinical chemistry, haematology or urinalysis result.

### Study design

**Figure 1:**
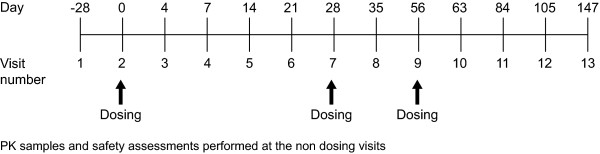
Schedule of study visits.

The study was a double-blind, randomised, placebo-controlled phase 1 study. Subjects attended the clinic for 13 visits over a 147-day study period (Figure 1). Subjects had a screening visit up to 28 days prior to randomisation. Study eligibility was confirmed at the randomisation visit, at which subjects were randomised to receive CAT-354 or placebo in addition to their usual medication (ICS and SABA). Subjects were recruited sequentially to one of three dose groups and randomised within each dose group to receive either CAT-354 (1 mg/kg, 5 mg/kg or 10 mg/kg) or placebo. Within each group, randomisation to CAT-354 or placebo was in a ratio of 4:1, respectively. CAT-354 was formulated at a nominal concentration of 10 mg/mL in phosphate-buffered saline (MedImmune). The placebo solution, phosphate-buffered saline, was supplied in matching vials. Study treatment was administered by 30-minute intravenous infusion at each of these visits using a syringe pump. Each subject received three doses of the assigned treatment, administered with a 28-day interval between doses. As a safety precaution, the first 3 subjects within each dose group were administered CAT-354 with at least 24 hours between subjects. Follow up for pharmacokinetic blood sampling and safety observations continued up to day 147 after the first dose (91 days after the third dose).

### Study procedures

#### Pharmacokinetic assessments

Serum samples for pharmacokinetic assessments were obtained pre-dose and at 10 minutes and 12 hours after the infusion on dosing days. Single samples were also collected at all other visits. Serum concentrations of CAT-354 and anti-CAT-354 were assessed by MedImmune using a quantitative sandwich ELISA based immunoassay performed on the Gyrolab assay platform. This method exhibits accuracy of ≤ 25% absolute relative error and precision of ≤ 20% coefficient of variation with a lower limit of quantification of 0.300 μg/mL.

#### Safety assessments

Safety was assessed by monitoring the occurrence of adverse events or abnormalities identified by standard laboratory tests, vital sign measurements, physical examinations and ECGs. Laboratory tests were performed at screening and 7 days after administration of each dose and the end of the study. Samples for urinalysis were collected at screening and the end of the study only, as were physical examinations. Vital signs were taken at screening and on dosing days pre-dose and prior to discharge after each dose and at the end of the study. ECGs were performed at the screening visit, within 6-7 hours after the first dose and at the end of the study.

#### Immunogenicity evaluations

To assess any immunogenic response, blood samples of all subjects taken pre-dose and at the last clinic visit were tested for the presence of anti-CAT-354 antibodies using a double-bridging ELISA assay [15] with a sensitivity of 1 μg/mL for the detection of an anti-idiotypic monoclonal antibody standard against CAT-354. Immuno-inhibition by CAT-354 and an isotype matched control antibody as an additional confirmatory assay was performed to eliminate false positive results.

#### Lung function assessments

FEV_1_, forced vital capacity (FVC) and the FEV_1_/FVC ratio were measured using a standardised, calibrated spirometer at each visit. At dosing visits, lung function was measured pre-dose and 30 minutes and 12 hours after the infusion. Wherever possible, pulmonary function tests were recorded at the same time of day.

### Statistical analysis

It was planned that eight subjects per dose group were to be evaluated for CAT-354. In addition, it was planned that a minimal number of subjects (6 in total) were to be allocated to placebo as a control for the assessment of tolerability (giving an overall ratio of 4:1 active:control). Cohorts of this size are considered to be adequate to provide the information required to fulfill the objectives of the study whilst exposing a minimum number of subjects to investigational product [16].

All safety and tolerability assessments were based on the safety population, defined as all subjects who received at least one dose of study material. No formal statistical testing was performed on safety data due to the exploratory nature of the study. The pharmacokinetic population was defined as all subjects in the safety population for whom sufficient post-dose blood samples were taken to estimate the observed maximum concentration (C_max_) and was used for pharmacokinetic parameter estimates. The pharmacokinetic parameters were estimated for each subject using WinNonlin software (Pharsight Corporation, California, version 5). Non-compartmental analysis was performed to generate parameter estimates using model 202 (intravenous infusion). The elimination phase volume of distribution (Vd) was calculated from clearance and the elimination rate constant.

## Results

### Demographics and baseline characteristics

**Figure 2:**
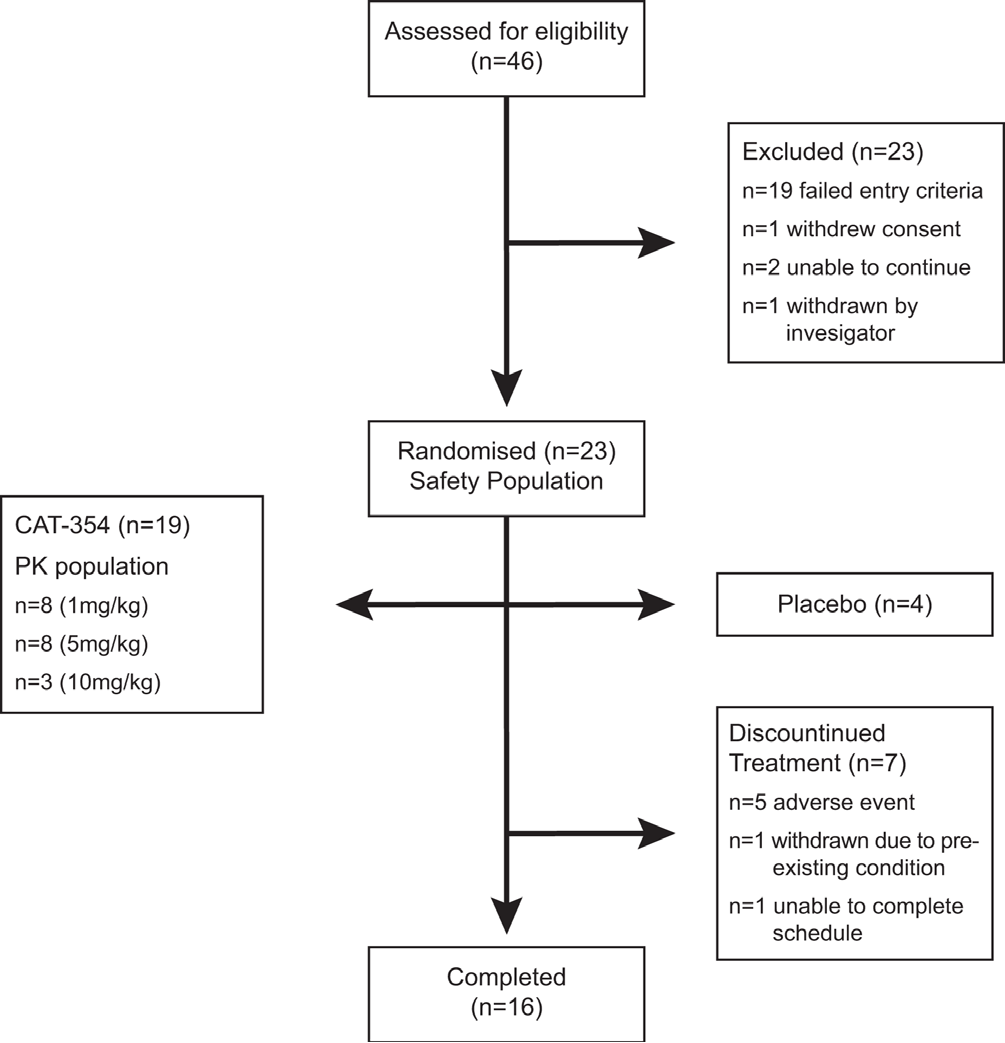
Flowchart of subjects through the study.

Due to the slow recruitment rate, the study was closed before the planned number of subjects was achieved in the highest dose group (10 mg/kg). A total of 23 subjects (22 male, 1 female) were randomised and included in the safety population. An additional 23 subjects were screened but were not eligible for the study (Figure 2). Of the 23 subjects included in the safety population, 19 subjects were also included in the pharmacokinetic population. Four subjects received placebo and were therefore not included in the pharmacokinetic population. Baseline characteristics of the safety population are shown in Table 1. All subjects had at least a 1-year history of asthma and were using ICS (mean overall beclomethasone dipropionate dose, 400 μg/day; range, 100-1000 μg/day) and SABA only.

**Table 1:** Baseline characteristics of the safety population

	Anti-IL-13			
		
	1 mg/kg (n = 8)	5 mg/kg (n = 8)	10 mg/kg (n = 3)	Placebo (n = 4)
Randomised, n	8	8	3	4
Completed study, n (%)	6 (75.0)	7 (87.5)	1 (33.3)	2 (50.0)
Sex, n (%)				
Male	8 (100)	8 (100)	2 (66.6)	4 (100)
Female	0	0	1 (33.3)	0
Age, years				
Mean (SD)	39.4 (9.1)	34.6 (7.6)	43.3 (14.5)	40.0 (13.0)
Range	26-54	21-46	34-60	26-53
Race, n (%)				
White	8 (100)	7 (87.5)	3 (100)	4 (100)
Black	0	1 (12.5)	0	0
Weight, kg				
Mean (SD)	78.5 (11.2)	88.3 (17.3)	78.3 (20.7)	76.0 (9.8)
BMI, kg/m^2^				
Mean (SD)	24.6 (3.0)	26.3 (4.3)	27.3 (3.1)	23 (3.0)
% predicted FEV_1_				
Mean (SD)	95.3 (8.3)	95.5 (9.8)	90.7 (9.5)	88.8 (4.6)
FEV_1_				
Mean (SD)	3.83 (0.54)	4.17 (0.54)	3.01 (1.35)	3.64 (0.68)
ICS dose, μg/d				
Median (range)	400 (200-400)	400 (200-1000)	200 (200-480)	

### Pharmacokinetics

**Figure 3:**
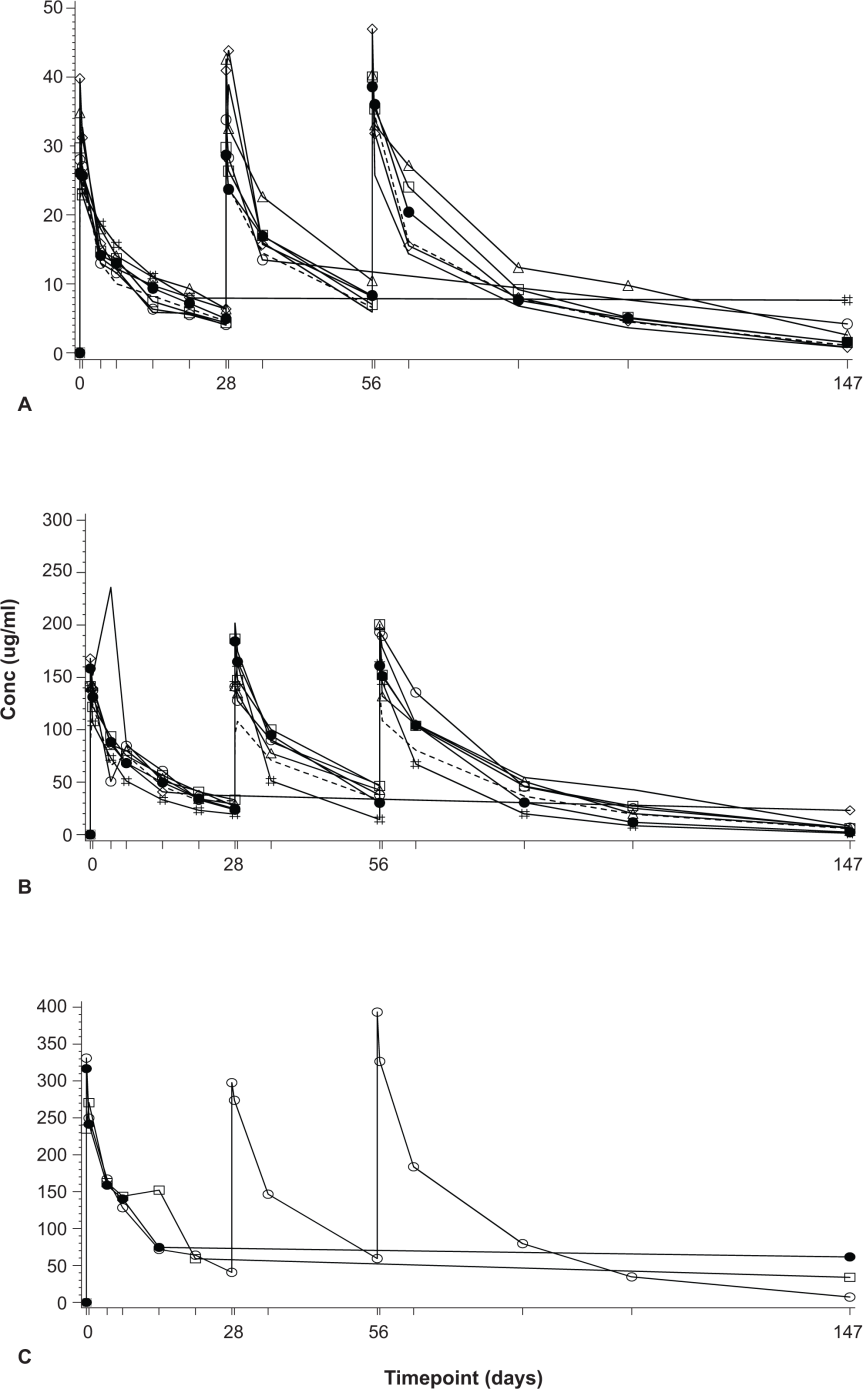
**Serum concentration versus time profile of multiple doses of CAT-354 at doses of (A) 1 mg/kg, (B) 5 mg/kg and (C) 10 mg/kg administered at 28-day intervals by intravenous infusion**.

Of the 19 subjects in the pharmacokinetic population, 5 did not complete the study and receive all three doses of CAT-354 (Figure 2). The full range of pharmacokinetic parameters could therefore only be derived for the 14 subjects who received all three doses. The serum concentration-time profiles for each dosing group are shown in Figure 3.

After a single dose, the half-life was 12-17 days and independent of dose level. Serum concentrations were still well above the limit of quantification by 28 days, when the next dose was given. Serum concentrations for the second and third doses generally increased at all time points.

Dose proportional increases in C_max_, AUC_(0-t) _and AUC_(0-inf) _were observed after a single dose (Table 2). Although assessment of the 10-mg/kg group was difficult due to the small sample size, the pharmacokinetics appeared to be proportional to those of lower doses (Table 2). Clearance (CL) (2.2-2.6 mL/day/kg) and volume of distribution (Vd) (44-57 mL/kg) were both low and independent of dose level. The half-life after the third dose was 14.8-25.1 days.

**Table 2:** Pharmacokinetic parameters after a single dose of CAT-354 (PK population)

	CAT-354		
	
	1 mg/kg (n = 8)	5 mg/kg (n = 8)	10 mg/kg (n = 3)
C_max _(μg/mL)	30.3 (5.2)	157 (34.5)	306 (31.4)
AUC(0-t) (μg.day/mL)	300 (42.0)	1691 (303)	3106 (719)
AUC(0-inf) (μg.day/mL)	428 (83.3)	2314 (393)	3861 (662)
t_1/2 _(days)	16.6 (2.7)	16.1 (3.6)	11.8 (1.9)
CL* (mL/day/kg)	2.41 (0.5)	2.23 (0.5)	2.64 (0.4)
Vd (mL/kg)	56.7 (8.0)	51.0 (10.4)	44.0 (0.8)

Note: Values are arithmetic mean (SD) after dose 1.

*Corrected for body weight

C_max _and the concentration after 28 days (C_28_) showed a small increase with multiple dosing (Table 3). These small increases were consistent with the calculated accumulation ratio (R_0_) for AUC_(0-t)_. The mean R_0 _was 1.58, 1.39 and 1.67 for 1, 5 and 10 mg/kg of CAT-354, respectively.

**Table 3:** Observed maximum concentration (C_max_) and concentration after 28 days (C_28_) following three doses of CAT-354 (PK population)

	CAT-354		
	
	1 mg/kg	5 mg/kg	10 mg/kg
C_max _(μg/mL)			
Dose 1	30.3 (5.2) [n = 8]	157 (34.5) [n = 8]	306 (31.4) [n = 3]
Dose 2	35.0 (6.8) [n = 7]	161 (32.8) [n = 7]	298 (-) [n = 1]
Dose 3	41.1 (4.8) [n = 6]	179 (25.4) [n = 7]	393 (-) [n = 1]
C_28 _(μg/mL)			
Dose 1	5.3 (1.3) [n = 8]	27.1 (4.9) [n = 7]	40.7 (-) [n = 1]
Dose 2	7.7 (1.6) [n = 6]	36.0 (11.5) [n = 7]	59.4 (-) [n = 1]
Dose 3	8.6 (2.0) [n = 6]	40.6 (12.3) [n = 7]	79.6 (-) [n = 1]

Note: Values are arithmetic mean (SD).

### Safety Profile

All 23 subjects experienced at least 1 adverse event. The majority of adverse events were of mild or moderate intensity and were not considered treatment-related (70 unrelated adverse events). The most common events were nasopharyngitis (reported by 17 subjects) and headache (reported by 10 subjects) and were reported both in subjects receiving CAT-354 and those receiving placebo (Table 4).

**Table 4:** Incidence of most common adverse events (safety population)

	No. (%) of Subjects [No. of Events]			
	
	CAT-354			
		
	1 mg/kg (n = 8)	5 mg/kg (n = 8)	10 mg/kg (n = 3)	Placebo (n = 4)
Nasopharyngitis	5 (62.5) [6]	7 (87.5) [10]	2 (66.7) [2]	3 (75.0) [4]
Headache	3 (37.5) [3]	4 (50.0) [6]	1 (33.3) [1]	2 (50.0) [4]
Lower respiratory tract infection	1 (12.5) [1]	1 (12.5) [1]	0	1 (25.0) [2]
Cough	1 (12.5) [1]	1 (12.5) [1]	0	1 (25.0) [1]
Pharyngolaryngeal pain	0	1 (12.5) [1]	1 (33.3) [2]	0
Pain	1 (12.5) [1]	1 (12.5) [1]	0	0
Chest discomfort	0	0	1 (33.3) [1]	1 (25.0) [1]
Diarrhoea	1 (12.5) [1]	1 (12.5) [1]	0	0

Adverse events that were considered by the investigator to be possibly related to treatment with CAT-354 were reported by three subjects. One subject experienced mild eczema that started 4 days after the second dose (1 mg/kg) and recovered after 6 weeks; 1 subject had a bloated stomach, epigastric discomfort and diarrhoea of moderate intensity on the day of the second dose (5 mg/kg) that lasted 2 days, plus moderate pain in the infusion arm that recovered on the same day; 1 subject experienced mild chest tightness on the day of dose 1 (10 mg/kg) that resolved on the same day.

Seven subjects were withdrawn from the study (Figure 2). One of these subjects was withdrawn after a chest infection that led to an exacerbation of asthma and was considered a serious adverse event. This occurred 20 days after the first dose (5 mg/kg) and was judged as unlikely to be related to the study drug. The subject recovered without obvious sequelae. Four additional subjects were withdrawn because of adverse events assessed by the investigator as unrelated or unlikely related to the study drug; 3 of these adverse events were respiratory tract infections. Two of these subjects received 1 mg/kg, 1 subject received 10 mg/kg and 1 subject received placebo. Two other subjects withdrew; one was withdrawn by the investigator because of the need for treatment of a pre-existing condition, and the other subject withdrew due to inability to complete the visit schedule.

There were no clinically significant effects for vital signs, ECG, laboratory or physical examination parameters. There was no evidence of dose-related changes in FEV_1_, FVC, FEV_1_/FVC ratio, and % predicted FEV_1 _of clinical concern and no increased use of rescue SABA except for the subject who had an exacerbation of asthma.

### Immunogenicity

There was no evidence of immunogenicity induced by CAT-354 in any of the samples collected from the subjects enrolled in this study.

## Discussion

This study has evaluated the pharmacokinetics and safety of CAT-354 at three dose levels administered by intravenous infusion on three occasions at intervals of 28 days. The pharmacokinetics were linear over the dose range studied. The half-life was found to be approximately 2-3 weeks, and the accumulation of drug was low. CAT-354 exhibited an acceptable safety profile with few adverse events that could have been related to treatment.

C_max _and AUC demonstrated a good relationship with dose level, whilst clearance and volume of distribution were independent of dose level, consistent with linear pharmacokinetics. The low clearance of CAT-354 (2.2-2.6 mL/day/kg) was consistent with the expected clearance of an IgG antibody that is not subject to target-mediated disposition [17]. The observed maximum concentration (C_max_) after each dose and 28 days after each dose (C_28_) increased by small amounts from dose 1 through dose 2 to dose 3 at all dose levels. The accumulation potential for CAT-354 was low with R_0 _not much greater than 1. However, with the observed pharmacokinetic characteristics, including low clearance, the drug remained at measurable concentrations in the serum, with levels still well above the quantifiable limit when final samples were taken up to 91 days after the final dose. The long half-life and low clearance of CAT-354 should maintain exposure to drug over the monthly dosing interval.

CAT-354 demonstrated an acceptable safety profile, with the majority of adverse events reported not related to study drug, and most events were of mild to moderate intensity. There were no dose-related adverse events and no subject experienced a serious adverse event that was thought to be related to study drug administration. There were two cases of diarrhoea post-infusion. Diarrhoea has been described as part of a constellation of symptoms that can occur during infusion reactions of mild intensity secondary to intravenous administration of antibodies [18]. These kinds of reactions, however, occurred at the lower doses of CAT-354, diminishing the likelihood of protein overload as a cause of the diarrhoea.

Seven subjects withdrew from the study, of which 5 were due to adverse events. Four of these adverse events were chest infections, with 3 occurring in the active treatment groups and 1 in the placebo group. It is to be expected that chest infections will occur in a multiple-dose study of long duration, and reassuringly there was no clear pattern for an increase in infections in the active treatment arms. The overall numbers of infections was low, and larger studies are needed to confirm the safety characteristics of this immune-modulating treatment. In addition, there were no effects of concern in any vital signs, ECG or laboratory parameters, and there were no effects of CAT-354 on lung function, which remained stable throughout the study treatment. There was no evidence of immunogenicity induced by CAT-354 in any of the samples collected during the study. These observations indicate that the doses of CAT-354 administered in the current study exhibited a safety profile suitable for further study. Future studies may also focus on the development of subcutaneous administration, as this will have practical advantages for administration.

There have been concerns about potential side effects of biological approaches to immunomodulation, such as the use of human monoclonal antibodies. The adverse event data, lung function, laboratory evaluations and immunogenicity tests in the current study suggest that CAT-354 can be safely administered in a repeat dosing schedule at doses up to 10 mg/kg. Although only 3 subjects received the 10-mg/kg repeat dose, no additional safety concerns were identified at this dose compared to the lower doses. Future studies could test repeated doses up to 10 mg/kg, and the next step for this drug would be to assess clinical efficacy such as through a properly powered study to evaluate pulmonary function. The current study used pulmonary function as a safety measure and was not properly powered to assess clinical benefits due to the low number of subjects.

The current study provides a safety assessment of multiple-dose administration of CAT-354 that supports further trials, and now efficacy studies are needed to evaluate the therapeutic potential of this drug. Pitrakinra is a recombinant IL-4 variant that competitively antagonises the IL-4Rα, and therefore interferes with the function of both IL-4 and IL-13 at the IL-4Rα/IL-13Rα1 receptor complex. This drug has been shown to inhibit the allergen challenge response in asthma [19]. The relative contributions of blocking IL-13 as opposed to IL-4 function to these results is not known, although animal studies suggest that IL-13 plays a dominant role in TH-2 inflammation [10-14]. Human proof-of-concept studies using CAT-354 are required to confirm these animal findings.

## Conclusions

The pharmacokinetics following repeat doses of 1 and 5 mg/kg CAT-354 were characterised in this study. The 10-mg/kg treatment group, although of a small sample size, indicated pharmacokinetics that were broadly proportional to lower doses. The safety profile of CAT-354 observed in this study warrants the conduct of future studies to evaluate clinical efficacy.

## Competing interests

The authors NAM, RF and LR are employees of MedImmune, LLC.

## Authors' contributions

DS, BK and AW were investigators for this study. RF and LR performed the pharmacokinetic analyses. NM participated in the design and coordination of this study. All authors read and approved the final manuscript.

## Pre-publication history

The pre-publication history for this paper can be accessed here:

http://www.biomedcentral.com/1471-2466/10/3/prepub
